# The efficacy and safety of intrathecal dexmedetomidine for parturients undergoing cesarean section: a double-blind randomized controlled trial

**DOI:** 10.1186/s12871-020-01109-4

**Published:** 2020-08-03

**Authors:** Xiao-xiao Li, Yu-mei Li, Xue-li Lv, Xing-he Wang, Su Liu

**Affiliations:** 1 Jiangsu Province Key Laboratory of Anesthesiology, Xuzhou Medical University, Xuzhou, Jiangsu China; 2 Feng Xian People’s Hospital of Jiangsu Province, Xuzhou, Jiangsu China; 3 Department of Anesthesiology, the Affiliated Hospital of Xuzhou Medical University, 99 Huaihai West Road, Xuzhou, 221000 Jiangsu China

**Keywords:** Intrathecal dexmedetomidine, Spinal anesthesia, Cesarean section

## Abstract

**Background:**

The efficacy and safety of spinal anesthesia by intrathecal dexmedetomidine (DEX) for parturients undergoing cesarean section are still lack of evidence. This aim of our study was to evaluate the efficacy and safety of intrathecal DEX for parturients undergoing cesarean section to provide more data evidence for intrathecal applications.

**Methods:**

Three hundred parturients undergoing cesarean section under spinal anesthesia were randomly assigned into three groups: group B: 9.0 mg (1.2 ml) of 0.75% bupivacaine with saline (1 ml); group FB: 9.0 mg (1.2 ml) of 0.75% bupivacaine with 20 μg of fentanyl (1 ml); group DB: 9.0 mg (1.2 ml) of 0.75% bupivacaine with 5 μg of DEX (1 ml). Intraoperative block characteristics, parturients’ postoperative quality of recovery, maternal and neonatal outcomes and the plasma concentration of DEX were measured. All parturients were followed up for 30 days to determine whether nerve injury occurred.

**Results:**

Compared with group B, the duration of sensory block in group FB and group DB were significantly prolonged (108.4 min [95% Confidence Interval (CI) = 104.6–112.3] in group B, and 122.0 min [95% CI = 116.8–127.3] in group FB, 148.2 min [95% CI = 145.3–151.1] in group DB). The overall score of quality recovery in group DB (71.6 [95% CI = 71.0–72.2]) was significantly higher than that in group FB (61.5 [95% CI = 60.8–62.2]) and group B (61.7 [95% CI = 61.0–62.4]). There was no statistically significant difference among the three groups for PH, PaO_2_, and PaCO_2_ of newborn. The plasma concentration of DEX in umbilical artery and umbilical vein was low and cannot be detected. The 30-days follow-up of parturients did not show any new onset of back, buttock or leg pain or paresthesia.

**Conclusions:**

DEX is a potential local anesthetic adjuvant that the intrathecal combination of 5 μg DEX can safely exhibit a facilitatory block effect and improve parturients’ recovery quality.

**Trial registration:**

Chinese Clinical Trial Registry (Registration number # ChiCTR1900022019; Date of Registration on March 20th, 2019).

## Background

Spinal anesthesia, with the advantage of easy-operating and avoiding the maternal risk of general anesthesia, including tracheal intubation failure, aspiration and lung infection, has been recommended as the preferred anesthesia for cesarean section [1–4]. However, some disadvantages caused by single-shot spinal anesthesia such us the limited duration of action and insufficient postoperative analgesia, which will lower the maternal postoperative recovery quality, and increasing local anesthetics doses is prone to cause maternal and neonatal adverse events [5, 6]. Therefore, several adjuvants [7, 8] in combination with local anesthetics have gradually been applicated to further improve spinal anesthesia, of which dexmedetomidine (DEX) is a good choice.

DEX, a highly selective α-2 adrenergic receptor agonist, provides sedative, analgesic, anti-sympathetic effects and has no significant effect on respiration [9]. Several clinical trials [10–13] have shown that DEX can be applicated as an auxiliary for spinal anesthesia through enhancing the anesthetic effects, preventing and reducing adverse reactions caused by local anesthetics. However, there are only a few studies on intrathecal DEX for cesarean section and these studies were mostly single-center with a small sample size, and whether the parturients’ recovery quality would be improved and whether DEX would adversely affect the fetus are still lack of plasma concentration evidence. Therefore, this two-centers, prospective, double-blind, randomized controlled trial was designed to evaluate the efficacy and safety of intrathecal DEX for parturients undergoing cesarean section to provide more data evidence for intrathecal applications.

## Methods

### Study participants

This trial was approved by the ethics committee of Feng Xian People’s Hospital and the Affiliated Hospital of Xuzhou Medical University. Written informed consent was obtained from all enrolled participants. This manuscript adheres to the applicable CONSORT guidelines. This study was a two-centers, prospective, double-blind, randomized controlled trial, and the two centers are the Affiliated Hospital of Xuzhou Medical University and Feng Xian People’s Hospital. Patient recruitment and data collection were started in April 2019 and ended in July 2019. The inclusion criteria of our study were: (1) Full-term pregnant women undergoing elective cesarean section under spinal anesthesia; (2) Age: 20 ~ 35 years; (3) ASA physical status II ~ III; The exclusion criteria were: (1) Multiple pregnancies; (2) Cardiovascular disease (e.g., pre-eclampsia and hypertension); (3) Serious hepatic dysfunction (Child-Pugh class C); (4) serious renal dysfunction (undergoing dialysis before surgery); (4) History of alcohol or opioid addiction; (5) Contraindication to spinal anesthesia; (7) Refusing to sign informed consent.

### Randomization, blinding and allocation concealment

According to the random number generated by computer, parturients were randomly allocated into three equal groups to receive either DEX, fentanyl or normal saline in combination with bupivacaine. The randomization sequence was placed in serially numbered opaque envelopes. Before the start of spinal anesthesia, an anesthesiologist prepared relevant drugs according to the randomization sequence and the anesthesiologist would not participate in the data collection, follow-up, and analysis.

### Study interventions

All parturients included in the study routinely fasted for 6–8 h before surgery, and none of them received pre-medication. When parturients were admitted into the operating room, standard monitoring for pulse oxygen saturation (SpO_2_), heart rate (HR), electrocardiogram (ECG), and noninvasive blood pressure (NIBP) was carried out. All parturients were given a supplementation of 3 L/min O_2_ through the nasal catheter. Then an intravenous 18-G cannula was inserted and patients were preloaded with ringer lactate 10 ml/kg 15–20 min before anesthesia.

With the parturients in the left lateral position, spinal anesthesia was performed at the L3-L4 interspace with a 25 G spinal Quincke-tip needle and study drugs were injected slowly within 15 s after the cerebrospinal fluid flowing out. The three groups were scheduled to receive drugs as follows: bupivacaine group (group B): 9 mg (1.2 ml) of 0.75% bupivacaine, with 1.0 ml of normal saline. Bupivacaine + fentanyl group: (group FB): 9 mg (1.2 ml) of 0.75% bupivacaine, with 20 μg of fentanyl in 1.0 ml of normal saline. Bupivacaine + DEX group (group DB): 9 mg (1.2 ml) of 0.75% bupivacaine, with 5 μg of DEX in 1.0 ml of normal saline. After removing the spinal needle, parturients were in the position with a 15-degree tilt to the left side immediately. All spinal anesthesia procedures were performed by experienced anesthesiologists. The sensory block level was tested by the pinprick method using a blunt 25-G needle. Assessment of the dermatomal level was done every 1 min until the peak sensory block level was achieved. Subsequently frequent testing every 10 min was performed until regression to S1 dermatome. The motor block was assessed by the modified Bromage scale (MBS, 0 = no paralysis,1 = inability to raise the leg, 2 = inability to flex the knee, and 3 = inability to flex the ankle) [14]. Surgery was allowed to commence once the sensory block level reached T6 [15]. Any patient showing moderate pain (visual analog score (VAS) ≥3) was administered intravenous 0.5 mg/kg ketamine. If hypotension (systolic blood pressure (SBP) < 90 mmHg or descending baseline values by 30%) persisted, intravenous 6 mg of ephedrine was administered; If bradycardia (HR < 50 bpm) occurs, intravenous 0.5 mg of atropine was administered. Repeat if necessary. Intraoperative ephedrine and atropine consumption were recorded. After surgery, all patients underwent patient-controlled intravenous analgesia (PCIA) with 2 μg/kg of sufentanil and 10 mg of tropisetron.

### Outcomes

The primary outcome of our study was the duration of sensory block, which was defined as time taken from intrathecal injection to sensory regression to S1 dermatome. The secondary outcomes of our study were as follows: the onset time of sensory block, which was defined as time taken from intrathecal injection to the maternal feeling of lower extremities temperature increment or numbness [16]; the onset time of motor block, which was defined as time taken from intrathecal injection to MBS > 1; the duration of motor block, which was defined as time taken from intrathecal injection to MBS = 0; the peak sensory block level; the blood gas analysis for PH, PaO_2_, and PaCO_2_ of the umbilical artery (UA) and umbilical vein (UV) blood samples of the newborn, which was performed immediately after collection; the plasma concentration of DEX in the UA and UV, which was determined by High-Performance Liquid Chromatography Tandem Mass Spectrometry methods [17]; Apgar scores, which were assessed at 1st and 5th min by the obstetrician who was blinded to the study; the hemodynamic parameters of parturient including BP, HR, which were evaluated at: baseline values (T0), immediately after blockcade (T1), 5 min (T2), 10 min (T3), 15 min (T4) and 20 min (T5) after blockcade. BP and HR at T0 were defined as the average values measured for 3 consecutive times at rest after entering the operating room.

The recovery quality of parturients within 24 h after surgery was assessed by obstetric quality of recovery-11 score [18] (ObsQoR-11, score from 0 to 10 in each term, where 0 = strongly agree and 10 = strongly disagree, the higher of the score, the higher of recovery quality), which was designed for parturients and presented by Ciechanowicz S; intra-and postoperative adverse events including nausea, vomiting and shivering, time to the first analgesic request and total sufentanil comsumption at 24 h after surgery were also recorded. Parturients were contacted by telephone for a post-operative 30 days following discharge to determine whether nerve injury occurred, including any new onset of back, buttock or leg pain or paresthesia. All of these evaluations were performed by an anesthesiologist blind to any other aspect of the trial.

### Statistical analysis

The sample size was calculated using PASS 15.0 software (NCSS, LLC, Kaysville, USA). The sample size calculation was based on the primary outcome, the duration of sensory block. According to our pilot trial results, the duration of sensory block was 114.3 ± 28.5 min for group B, 120.1 ± 29.4 min for group FB, 128.8 ± 29.5 min for group DB. A total of 80 patients were required to achieve 80% power with an alpha error of 5% based on the module of analysis of variance (ANOVA) in PASS. Considering a lost-to-follow-up rate of about 15%, 94 patients are required for each group. Finally, a total of 100 parturients were recruited in our study.

Statistical analysis was performed using IBM SPSS 22.0 software (SPSS Inc., IBM, Chicago, IL, USA). Numeric variables were analyzed for normality by the Kolmogorov-Smirnov test. Normally distributed continuous variables were expressed as mean with a standard deviation and compared using ANOVA with post hoc analysis using Bonferroni test. The categorical variables were presented as number (%) and compared using Chi-square test or Fischer exact test. Kaplan-Meier curve illustrated the time to first analgesic request and comparisons between groups were conducted with the log-rank test. Hemodynamic parameters were compared by repetitive measurement deviation analysis. *P* < 0.05 was considered statistically significant.

## Results

**Fig. 1:**
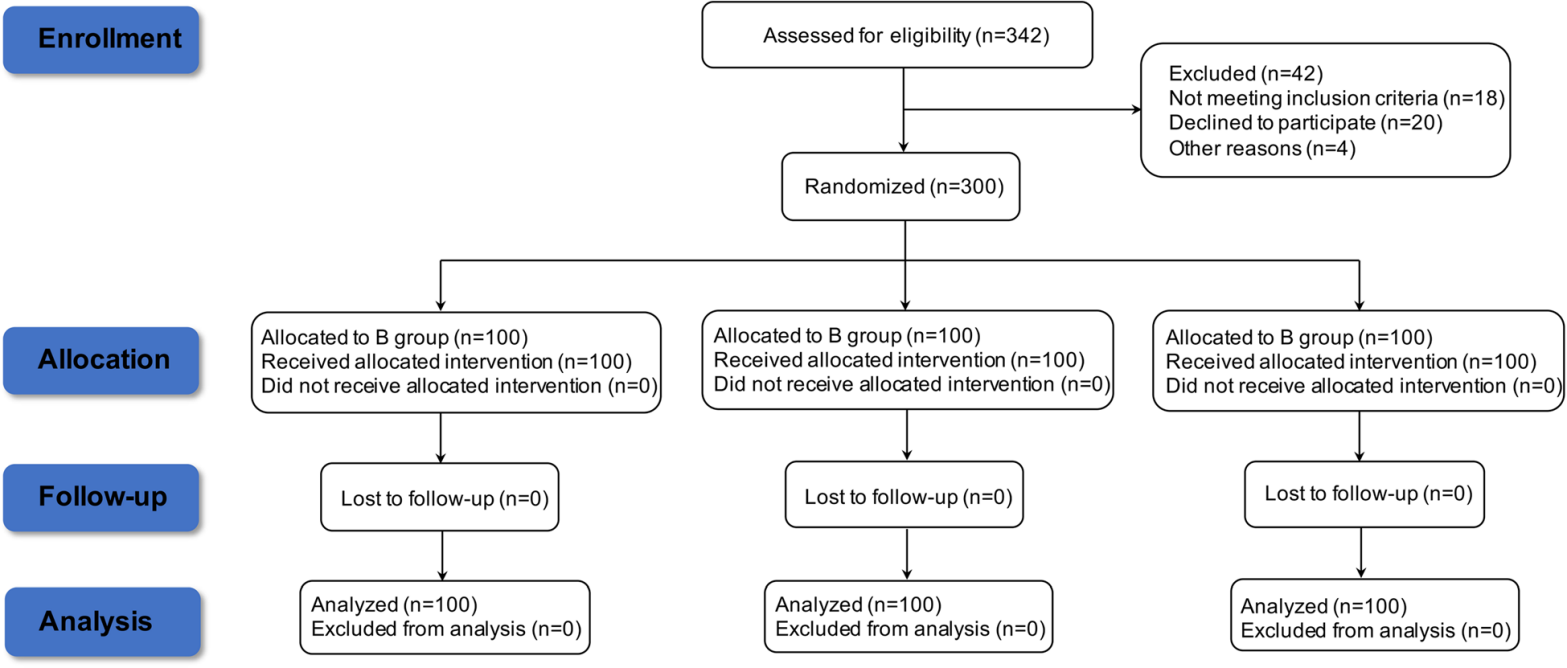
Study population flow diagram

Between April and July in 2019, 342 pregnant women at two centers were evaluated for study participation. Of these, eighteen women did not meet the inclusion criteria, twenty women refused to participate, and four women were excluded for other reasons (Fig. 1). Finally, three hundred patients were randomly 1:1:1 divided into group B (*n* = 100), group FB (*n* = 100) and group DB (*n* = 100). All patients were well-blocked and no one needed additional analgesia during the surgery. In addition, all patients completed the assessment and received postoperative follow-up for 30 days**.**

The three groups were comparable with regard to baseline variables include age, height, weight, BMI, ASA physical status, gestational age. There were also no significant differences in perioperative variables including peak sensory level, duration of surgery, intraoperative fluid volume and blood loss (Table 1).

**Table 1:** Baseline and perioperative characteristics of parturients

	Group B (*n* = 100)	Group FB (*n* = 100)	Group DB (*n* = 100)	*P-*Value
Age (yr.)	27 (26–29)	27 (25–30)	27 (25–29)	0.244
Height (cm)	161.5 (159.0–164.0)	162.0 (159.0–165.0)	160 (159.0–164.0)	0.284
Weight (kg)	72.3 ± 6.2	72.8 ± 5.3	72.6 ± 5.3	0.771
BMI (kg/m^2^)	27.6 ± 2.5	27.6 ± 2.2	27.8 ± 2.3	0.865
ASA physical status, n (%)				0.394
II	78 (78)	74 (74)	82 (82)	
III	22 (22)	26 (26)	18 (18)	
Gestational week (Wk.)	39 (38–39)	39 (38–39)	39 (38–39)	0.379
Peak sensory level				0.846
T2	2 (2)	1 (1)	1 (1)	
T4	37 (37)	36 (36)	42 (42)	
T6	61 (61)	63 (63)	57 (57)	
Surgery duration (min)	41.0 (38.0–44.0)	41.0 (39.0–44.0)	41.0 (39.0–44.0)	0.746
Intraoperative fluid volume (ml)	1351.1 ± 115.4	1361.6 ± 97.8	1356.5 ± 98.7	0.775
Intraoperative blood loss (ml)	421.2 ± 36.0	424.5 ± 30.5	423.1 ± 30.7	0.773

**Notes:** Data are presented as n (%) or mean ± SD or median (range); There were no significant differences among the three groups (*P* > 0.05). Group B = bupivacaine group; Group FB = bupivacaine and fentanyl group; Group DB = bupivacaine and dexmedetomidine group

Abbreviations: *ASA* American Society of Anesthesiologists, *BMI* Body Mass Index

**Fig. 2:**
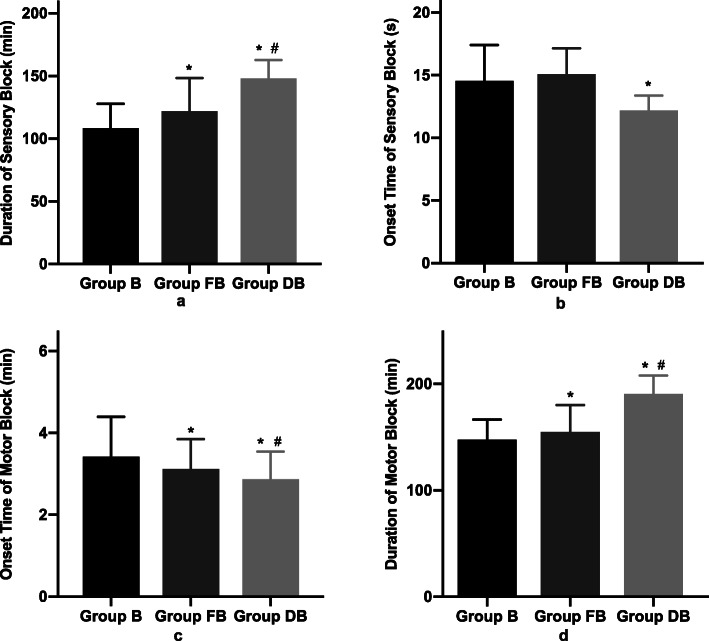
Block characteristics of parturients Notes: Group B = bupivacaine group; Group FB = bupivacaine and fentanyl group; Group DB = bupivacaine and dexmedetomidine group. * *P* < 0.017 Group DB or Group FB vs Group B; ^#^*P* < 0.017 Group DB vs Group FB.

Compared with group B, the duration of sensory block in group FB and group DB were prolonged (108.4 min [95% Confidence Interval (CI) = 104.6–112.3] in group B, and 122.0 min [95% CI = 116.8–127.3] in group FB, 148.2 min [95% CI = 145.3–151.1] in group DB) with statistical significance (*P* < 0.001) (Fig. 2a). The duration of sensory block was significantly longer in group DB as compared with group FB (*P* < 0.001). Compared with group B, the onset time of sensory block (Fig. 2b) in group DB was significantly shorter (12.2 s [95% CI = 12.0–12.4]) in group DB, 14.5 s [95% CI = 14.0–15.1] in group B, *P* < 0.001). Besides, compared with group B and group FB (Fig. 2c), the onset time of motor block in group DB was statistically shorter (2.9 min [95% CI = 2.7–3.0] in group DB, 3.1 min [95% CI = 3.0–3.3] in group FB, 3.4 min [95% CI = 3.2–3.6], in group B, *P* < 0.001). However, compared with group B (147.5 min [95% CI = 143.7–151.3]), the duration of motor block (Fig. 2d) in group DB (190.3 min [95% CI = 186.9–193.8]) was prolonged by 43 min (*P* < 0.001), while that in group FB (154.9 min [95% CI = 150.0–160.0]) was prolonged by 7 min (*P* = 0.038).

There were 11 items in ObsQoR-11 score Table (Table 2) to reflect the quality of postoperative recovery. The overall score of group DB (71.6 [95% CI = 71.0–72.2]) was higher than that of group FB (61.5 [95% CI = 60.8–62.2], *P* < 0.001) and group B (61.7 [95% CI = 61.0–62.4], *P* < 0.001). All items showed recovery quality of group DB was significantly better than that of group B, except in terms of feeling dizzy (*P* > 0.05). Moreover, there was no statistical difference between group B and group FB about the ObsQoR-11 score.

**Table 2:** ObsQoR-11 of parturients

	Group B (*n* = 100)	Group FB (*n* = 100)	Group DB (*n* = 100)	*P-*Value
Moderate pain	3.6 ± 1.1	5.6 ± 0.9*	7.3 ± 1.2*^#^	< 0.001
Severe pain	4.4 ± 1.2	5.0 ± 2.1*	7.5 ± 1.6*^#^	< 0.001
Nausea or vomiting	5.2 ± 1.0	6.0 ± 0.9*	7.3 ± 1.2*^#^	< 0.001
Feeling dizzy	6.3 ± 1.2	5.0 ± 0.8*	6.3 ± 1.1^#^	< 0.001
Shivering	3.7 ± 0.8	5.3 ± 0.7*	7.2 ± 1.0*^#^	< 0.001
Have been comfortable	6.4 ± 1.0	6.4 ± 0.6	7.4 ± 1.8*^#^	< 0.001
Able to mobilize independently	6.0 ± 1.9	6.9 ± 1.5*	6.9 ± 1.1*	< 0.001
Can hold baby without assistance	6.9 ± 0.8	8.2 ± 0.7*	8.1 ± 0.8*	< 0.001
Can feed/nurse baby without assistance	6.6 ± 0.7	7.0 ± 1.0*	7.1 ± 0.7*	< 0.001
Can look after personal hygiene/toilet	5.6 ± 0.9	6.3 ± 0.6*	6.5 ± 0.9*	< 0.001
Feeling in control	7.0 ± 0.8	7.3 ± 1.0*	8.0 ± 0.8*^#^	< 0.001
Total	61.7 ± 3.3	61.5 ± 3.6	71.6 ± 3.1*^#^	< 0.001

Notes: Data are presented as mean ± SD; Group B = bupivacaine group; Group FB = bupivacaine and fentanyl group; Group DB = bupivacaine and dexmedetomidine group; ObsQoR-11 = obstetric quality of recovery-11 score, 0–10 in each term, where 0 = strongly agree and 10 = strongly disagree

* *P* < 0.017 Group DB or Group FB vs Group B; ^#^*P* < 0.017 Group DB vs Group FB

**Fig. 3:**
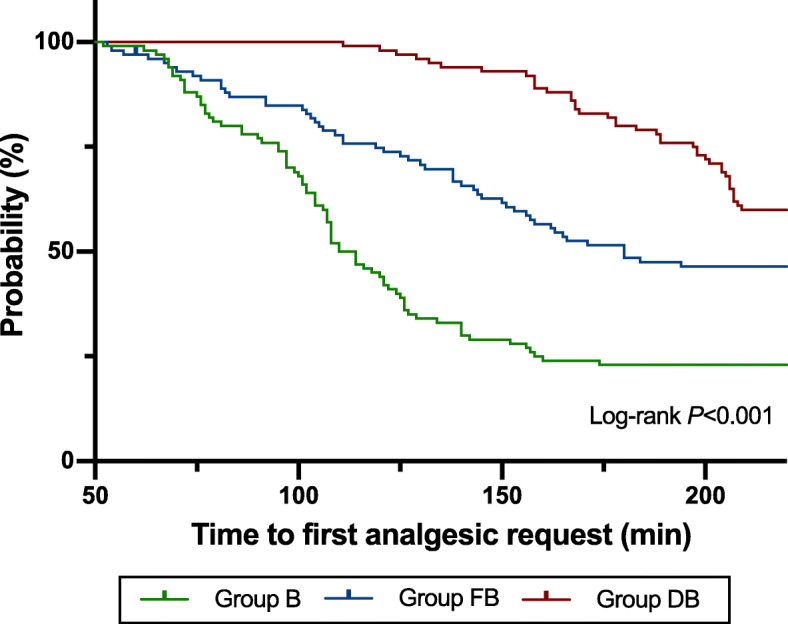
Kaplan-Meier curves for time to first analgesic request. Notes: Group B = bupivacaine group; Group FB = bupivacaine and fentanyl group; Group DB = bupivacaine and dexmedetomidine group

Kaplan-Meier curve (Fig. 3) showed that time to first analgesic request in group DB was longer than that in group FB and group B (log-rank *P* < 0.017). However, the sufentanil dosage within postoperative 24 h was not statistically different among three groups (*P* = 0.681).

**Fig. 4:**
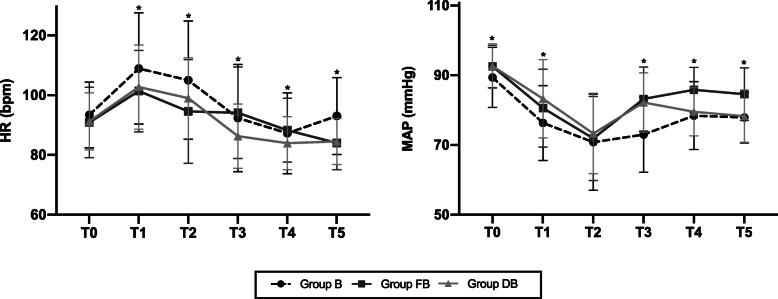
Hemodynamic parameters. Notes: Group B = bupivacaine group; Group FB = bupivacaine and fentanyl group; Group DB = bupivacaine and dexmedetomidine group; T0 = before spinal anesthesia, T1, T2, T3, T4, T5 = 0, 5, 10, 15, 20 min after spinal anesthesia. HR = Heart Rate; MAP = Mean Arterial Pressure; *There were significant differences among the three groups (*P* < 0.05)

The maternal hemodynamic characteristics including HR and mean arterial pressure (MAP) were found significantly higher in group DB than that in group B (Fig. 4). The incidence of shivering (Table 3) was statistically lowered in group DB (3%) compared with group FB (18%) and group B (35%). The incidence of hypotension in group DB (33%) was higher than that in group FB (25%) and group B (28%) but with no statistical difference. There was no statistical difference for the dosage of ephedrine and atropine, intra-operative or post-operative nausea and vomiting among three groups.

**Table 3:** Maternal outcomes

	Group B (*n* = 100)	Group FB (*n* = 100)	Group DB (*n* = 100)	*P*-Value
Shivering, n (%)	35 (35)	18 (82) *	3 (97) *^#^	< 0.001
Hypotension, n (%)	28 (28)	25 (25)	33 (33)	0.450
Dose of ephedrine (mg)	2.8 ± 3.9	2.1 ± 3.6	1.5 ± 3.0	0.029
Nausea and/or vomiting, n (%)	11 (11)	17 (17)	14 (14)	0.474
Sufentanil consumption (μg)	106.0 ± 9.2	105.5 ± 7.7	105.0 ± 7.8	0.681

Notes: Data are presented as n (%) or mean ± SD; Group B = bupivacaine group; Group FB = bupivacaine and fentanyl group; Group DB = bupivacaine and dexmedetomidine group

*P < 0.017 Group DB or Group FB vs Group B; ^#^*P* < 0.017 Group DB vs Group FB

For PH, PaO_2_, and PaCO_2_ in the umbilical artery and umbilical vein blood of newborn (Table 4), there were no statistically significant among the three groups. The concentration of DEX in umbilical artery and umbilical vein was too low to be detected by High-Performance Liquid Chromatography Tandem Mass Spectrometry. The mean values of Apgar scores at 1st and 5th min were all beyond 8, which also showed no statistical significance. Moreover, the 30-daysfollow-up did not show any new onset of back, buttock or leg pain or paresthesia.

**Table 4:** Neonatal outcomes

	Group B (*n* = 100)	Group FB (*n* = 100)	Group DB (*n* = 100)	*P*-Value
Umbilical artery				
pH	7.3 ± 0.3	7.3 ± 0.2	7.3 ± 0.4	0.581
PaO_2_ (mmHg)	15.6 ± 2.0	15.0 ± 2.3	15.4 ± 2.4	0.217
PaCO_2_ (mmHg)	49.9 ± 3.4	49.8 ± 4.0	50.4 ± 4.6	0.545
Umbilical vein				
pH	7.4 ± 0.2	7.4 ± 0.1	7.4 ± 0.5	0.711
PaO_2_ (mmHg)	30.0 ± 3.4	30.7 ± 4.4	30.80 ± 3.5	0.277
PaCO_2_ (mmHg)	42.6 ± 3.2	41.6 ± 3.8	42.0 ± 3.2	0.111
Apgar score				
1 min	8.7 ± 0.5	8.7 ± 0.5	8.7 ± 0.5	0.752
5 min	9.7 ± 0.5	9.7 ± 0.4	9.7 ± 0.5	0.809

Notes: Data are presented as mean ± SD; Group B = bupivacaine group; Group FB = bupivacaine and fentanyl group; Group DB = bupivacaine and dexmedetomidine group

*P < 0.017 Group DB or Group FB vs Group B; ^#^*P* < 0.017 Group DB vs Group FB

## Discussion

Our results showed that compared with 9 mg of bupivacaine alone, the combination of 5 μg of DEX for cesarean section could significantly prolong the duration of sensory block and improve paturients’ recovery quality with no neonatal adverse effects or maternal neurotoxicity in the short term.

Spinal anesthesia, which is block-well, easy to operate, not as complicated as epidural anesthesia [19], and avoiding the maternal risk of general anesthesia, has become the preferred anesthesia type for cesarean section. However, in clinical practice, single-shot spinal anesthesia was often not sufficient to inhibit visceral pain, causing maternal discomfort during the surgery, which affect parturients’ postoperative recovery quality [6]. While increasing the doses of local anesthetics to prolong the analgesic time could lead to adverse effects such as central nervous system problems and cardiotoxicity. In our study, compared with intrathecal 9 mg of bupivacaine alone, the onset time of sensory and motor block of parturients in combination of 9 mg of intrathecal bupivacaine with 5 μg of DEX was significantly shortened, and the duration of sensory block was significantly prolonged by 40 min, which is consistent with the research results of Suthar’s [20] and Sushruta’s [10]. The mechanism may be as follows: DEX can activate the α-2 adrenergic receptor in the dorsal horn neurons, activate the spinal cord intermediate neurons by reducing the neurotransmitter released by the primary afferent end and G-protein-mediated potassium channel, and make the spinal cord intermediate neurons hyperpolarized, thus reducing the pain transmission. In addition, DEX can also block the internal flow of Na + and enhance the blocking effect of local anesthetics on the sodium channel of the cell membrane [21, 22]. However, consisted with the results of a meta-analysis [23] that included 9 RCTs, our study found that motor block duration of paturients with intrathecal DEX was also prolonged, which suggest that combination with DEX may increase the fall risk and delay the early rehabilitation of parturients.

Currently, the commonly used postoperative recovery quality scales were QoR-40 [24] and QoR-15 [25]. However, both of them are developed and verified in non-obstetric patients and day surgery population [26], so there are many items unrelated to cesarean section, and lack of critical elements to evaluate postoperative recovery after delivery such as the ability of caring babies [18]. The ObsQoR-11 scale has been proved to be reliable, clinically acceptable, feasible and effective in patients undergoing elective and emergency cesarean section [18, 27]. In our study, all questionnaire feedback had been received and the results showed that scores in group DB was higher than in both group FB and group B (*P* < 0.017), suggesting that parturients with intrathecal DEX had a better recovery quality.

Consistent with the findings of meta-analysis conducted by Miao [12], intrathecal DEX can significantly reduce the incidence of shivering in parturients undergoing spinal anesthesia. The mechanism of anti-shivering effect can be inferred that DEX could reduce central thermos-sensitivity by weakening the electrical conductivity of neurons through mediating the α-2 adrenergic receptors in the brain and spinal cord [28, 29]. Moreover, intrathecal DEX showed added advantages on block characteristics and ObsQoR-11 score compared with intrathecal fentanyl, suggesting a better clinical application prospect.

When intrathecal DEX during cesarean section, one of the main concerns was the maternal neurotoxicity. Therefore, all participants were followed up for 30 days after surgery, and none of them showed neurological complications of lower limbs and buttocks, indicating that intrathecal DEX would not lead to nerve injury in the short term. Ozdamar [30] injected 10 rats with DEX 10 μg through the subarachnoid path and extracted spinal medulla for histological and electron microscopy examination after 7 days, and the results showed that compared with saline group, no signs of neuronal or axonal injury, gliosis, or myelin sheath damage was found. Another concern was the adverse effects on the fetus, which was excluded by the blood gas analysis and Apgar scores in our study. Li et al. [31] showed similar results, which further confirmed our conclusion. A placental perfusion study in vitro conducted by Ala-Kokko [32] found that the DEX fetal: maternal concentration ratio was 0.77, which meant DEX in maternal circulation was easy to pass through the placental barrier. Currently, there is no firm clinical data about whether DEX would be absorbed into the maternal circulation and transferred to the fetus via the placenta under the intrathecal administration. In our study, the plasma concentration of DEX in the UA and UV was measured and no DEX accumulation was detected, suggesting that intrathecal 5 μg of DEX caused a low or even no drug exposure on the fetus, which would not lead to adverse effects.

There were also some limitations in our study. Firstly, the adequacy of muscle relaxation during the surgery and the satisfaction of parturients and obstetricians were not measured, further studies should use more parameters to explore the efficacy of intrathecal DEX; Secondly, due to the invasive arterial blood pressure monitoring was not performed during the operation, the information concerning the placental transfer was not obtained with no maternal blood plasma sample collected; Thirdly, we did not investigate the dose-response reaction of DEX, and the optimal clinical dose was not determined. Furthermore, the postoperative follow-up period in this study was only 30 days, so it is unknown whether patients had delayed adverse neuron reactions.

## Conclusion

DEX is a potential local anesthetic adjuvant that the intrathecal combination of 5 μg DEX can safely exhibit a facilitatory block effect and improve parturients’ recovery quality. However, large sample clinical studies to support the safety of intrathecal DEX use in the clinical setting are still needed.

## Supplementary information

**Additional file 1:.** Table S1 Block characteristics.

## Data Availability

The datasets generated and/or analyzed during the current study are available from the corresponding author on reasonable request.

## Abbreviations

DEX: Dexmedetomidine
CI: Confidence Interval;
SpO2: Pulse Oxygen Saturation
HR: Heart Rate
ECG: Electrocardiogram
NIBP: Noninvasive Blood Pressure
SBP: Systolic Blood Pressure
MBS: Modified Bromage scale
ObsQoR-11: Obstetric Quality of Recovery-11 Score
MAP: Mean Arterial Pressure
RR: Relative Risk
ASA: American Society of Anesthesiologists
BMI: Body Mass Index
