# Supplementary material for: The efficacy and safety of intrathecal dexmedetomidine for parturients undergoing cesarean section: a double-blind randomized controlled trial
Source: BMC Anesthesiol. 2020 Aug 3;20:190. doi: 10.1186/s12871-020-01109-4 (PMC7397624; doi:10.1186/s12871-020-01109-4)
Supplement: Supplementary file 1 — Additional file 1:. Table S1 Block characteristics. [file 12871_2020_1109_MOESM1_ESM.docx]

**Additional Table 1** Block characteristics.

| Characteristic | Group B (n=100) | Group FB (n=100) | Group DB (n=100) | *P*-Value | |
| --- | --- | --- | --- | --- | --- |
| Sensory Block | | | | |  |
| Onset time (s) | 14.5±2.9 | 15.1±2.1 | 12.2±1.2*^#^ | <0.001 |  |
| Duration (min) | 108.4±19.3 | 122.0±26.4* | 148.2±14.6*^#^ | <0.001 |  |
| Motor Block | | | | |  |
| Onset time (min) | 3.4±1.0 | 3.1±0.7* | 2.9±0.7*^#^ | <0.001 |  |
| Duration (min) | 147.5±19.2 | 154.9±25.2* | 190.3±17.4*^#^ | <0.001 |  |

**Notes:** Group B = bupivacaine group; Group FB = bupivacaine and fentanyl group; Group DB = bupivacaine and dexmedetomidine group.

* *P*<0.017 Group DB or Group FB vs Group B; ^#^ *P*<0.017 Group DB vs Group FB.
